# Upregulation of C_4_ characteristics does not consistently improve photosynthetic performance in intraspecific hybrids of a grass

**DOI:** 10.1111/pce.14301

**Published:** 2022-03-10

**Authors:** Matheus E. Bianconi, Graciela Sotelo, Emma V. Curran, Vanja Milenkovic, Emanuela Samaritani, Luke T. Dunning, Lígia T. Bertolino, Colin P. Osborne, Pascal‐Antoine Christin

**Affiliations:** ^1^ Ecology and Evolutionary Biology, School of Biosciences University of Sheffield Western Bank Sheffield UK; ^2^ Plants, Photosynthesis and Soil, School of Biosciences University of Sheffield Western Bank Sheffield UK

**Keywords:** C_3_–C_4_ intermediates, C_4_ photosynthesis, complex traits, hybrids, leaf anatomy, Poaceae, transcriptome

## Abstract

C_4_ photosynthesis is thought to have evolved via intermediate stages, with changes towards the C_4_ phenotype gradually enhancing photosynthetic performance. This hypothesis is widely supported by modelling studies, but experimental tests are missing. Mixing of C_4_ components to generate artificial intermediates can be achieved via crossing, and the grass *Alloteropsis semialata* represents an outstanding study system since it includes C_4_ and non‐C_4_ populations. Here, we analyse F1 hybrids between C_3_ and C_4_, and C_3_+C_4_ and C_4_ genotypes to determine whether the acquisition of C_4_ characteristics increases photosynthetic performance. The hybrids have leaf anatomical characters and C_4_ gene expression profiles that are largely intermediate between those of their parents. Carbon isotope ratios are similarly intermediate, which suggests that a partial C_4_ cycle coexists with C_3_ carbon fixation in the hybrids. This partial C_4_ phenotype is associated with C_4_‐like photosynthetic efficiency in C_3_+C_4_ × C_4_, but not in C_3_ × C_4_ hybrids, which are overall less efficient than both parents. Our results support the hypothesis that the photosynthetic gains from the upregulation of C_4_ characteristics depend on coordinated changes in anatomy and biochemistry. The order of acquisition of C_4_ components is thus constrained, with C_3_+C_4_ species providing an essential step for C_4_ evolution.

## Summary statement

We analyse hybrids between C_3_ and C_4_, and C_3_+C_4_ and C_4_ accessions of the grass *Alloteropsis semialata*. While hybrids have upregulated C_4_ features, these do not consistently increase photosynthetic efficiency, highlighting the importance of the order of acquisition of components during C_4_ evolution.

## 1 INTRODUCTION

C_4_ photosynthesis is a complex adaptation that allows plants to sustain high photosynthetic rates in conditions that limit CO_2_ availability in the leaf, such as hot, dry and saline environments (Sage, 2004). The C_4_ metabolism relies on a biochemical cycle operating within a specialized leaf anatomy, which concentrates CO_2_ around Rubisco, suppressing the energetically costly photorespiratory pathway (Hatch, 1987). The C_4_ trait is highly polyphyletic, but its multiple origins are mostly clustered in a few groups (Sage et al., 2011). The increased evolutionary accessibility to a C_4_ physiology in these groups has been associated with a number of ecological, anatomical and genetic features that acted as evolutionary enablers (Christin et al., 2013; Edwards, 2019; Moreno‐Villena et al., 2018; Sage, 2001). The classic model of C_4_ evolution hypothesizes that, once these enablers are in place, an intermediate metabolism that relies on a photorespiratory CO_2_ pump is established (in ‘type I' C_3_–C_4_ intermediates or ‘C_2_' plants), and gradually complemented by a weak C_4_ cycle (in ‘type II' C_3_–C_4 _sensu Edwards & Ku, 1987, or ‘C_3_+C_4_' plants sensu Dunning et al., 2017), which is subsequently optimized (Hylton et al., 1988; Monson & Moore, 1989; Sage, 2004). The transition through the intermediate stages has been predicted by mechanistic modelling studies, which support the idea that the sequential acquisition of components of the C_4_ trait successively increases fitness through higher photosynthetic output (Heckmann et al., 2013, 2016; Mallmann et al., 2014). This model however currently lacks experimental support. Efforts to engineer the C_4_ trait into C_3_ crops have provided the first opportunities to study the effects of C_4_ components in isolation (Ermakova et al., 2021; Ishimaru et al., 1997; Taniguchi et al., 2008; Wang et al., 2017), but the recipient species do not necessarily possess the anatomical properties required for an efficient C_4_ metabolism. An alternative strategy is to segregate the components of the C_4_ trait via crosses between C_4_ and non‐C_4_ plants (Simpson et al., in press).

Successful interspecific crosses between C_4_ and non‐C_4_ plants have been reported particularly for the eudicot genera *Atriplex* (e.g., Björkman et al., 1969; Oakley et al., 2014) and *Flaveria* (e.g., Araus et al., 1990; Byrd et al., 1992; reviewed by Brown & Bouton, 1993), with a recent interest in exploring this strategy to dissect the C_4_ trait (Lin et al., 2021; Oakley et al., 2014; Simpson et al., in press; Sultmanis, 2018). Most of the diversity of C_4_ lineages however lies in monocots, and especially the grass family, with at least 22 independent origins of C_4_ photosynthesis (Grass Phylogeny Working Group II [GPWG II], 2012). The only existing crosses between photosynthetic types in monocots concerned C_3_ and C_3_–C_4_ parents (Bouton et al., 1986), and the grass *Alloteropsis semialata* offers a particularly suitable system to complement these studies, as it includes not only C_3_ and C_4_ (Ellis, 1974a), but also C_3_+C_4_ individuals (Lundgren et al., 2016). While different photosynthetic types of *A. semialata* can hybridize (Bianconi et al., 2020), the effects of different crosses remain to be described. *Alloteropsis semialata* has a paleotropical distribution, with C_3_ and C_3_+C_4_ populations in Southern and Central/Eastern regions of Africa, respectively, and C_4_ individuals occurring across Africa, South and Southeast Asia, and Oceania (Bianconi et al., 2020; Lundgren et al., 2015; Olofsson et al., 2016, 2021). The photosynthetic types in *A. semialata* are associated with distinct genetic lineages (Lundgren et al., 2015; Olofsson et al., 2016), which initially diverged around 3 Mya (Bianconi et al., 2020). Although the ranges of C_4_ and non‐C_4_ lineages geographically overlap in some regions of Africa, with C_3_+C_4_ and C_4_ in Central/Eastern Africa, and C_3_ and C_4_ in Southern Africa, genetic analyses have shown that natural hybrids are rare (Bianconi et al., 2020; Olofsson et al., 2016, 2021). The lack of a clear hybrid zone has been partially explained by the differences in ploidy levels among photosynthetic types where they are found in sympatry (Olofsson et al., 2021). However, the broad ranges of C_4_ and C_3_+C_4_ diploids overlap, and they have been coexisting for at least one million years (Bianconi et al., 2020; Lundgren et al., 2015). Besides ploidy differences, the low frequency of natural hybridization might be associated with pre‐ and postzygotic barriers, such as possible hybrid depression associated with pleiotropic costs of upregulating components of C_4_ photosynthesis without a fully functional C_4_ metabolism in place (Olofsson et al., 2021). Experimental crosses between C_4_ and non‐C_4_ individuals of *A. semialata* provide an opportunity to test this hypothesis.

*Alloteropsis semialata* has a paleotropical distribution, with C_3_ and C_3_ + C_4_ populations in Southern and Central/Eastern regions of Africa, respectively, and C_4_ individuals occurring across Africa, South and Southeast Asia, and Oceania (Bianconi et al., 2020; Lundgren et al., 2015; Olofsson et al., 2016, 2021). The photosynthetic types in *A. semialata* are associated with distinct genetic lineages (Lundgren et al., 2015; Olofsson et al., 2016), which initially diverged around 3 Mya (Bianconi et al., 2020). Although the ranges of C_4_ and non‐C_4_ lineages geographically overlap in some regions of Africa, with C_3_ + C_4_ and C_4_ in Central/Eastern Africa, and C_3_ and C_4_ in Southern Africa, genetic analyses have shown that natural hybrids are rare (Bianconi et al., 2020; Olofsson et al., 2016, 2021). The lack of a clear hybrid zone has been partially explained by the differences in ploidy levels among photosynthetic types where they are found in sympatry (Olofsson et al., 2021). However, the broad ranges of C_4_ and C_3_ + C_4_ diploids overlap, and they have been coexisting for at least one million years (Bianconi et al., 2020; Lundgren et al., 2015). Besides ploidy differences, the low frequency of natural hybridization might be associated with pre‐ and postzygotic barriers, such as possible hybrid depression associated with pleiotropic costs of upregulating components of C_4_ photosynthesis without a fully functional C_4_ metabolism in place (Olofsson et al., 2021). Experimental crosses between C_4_ and non‐C_4_ individuals of *A. semialata* provide an opportunity to test this hypothesis.

Here we generate hybrids between C_3_ and C_4_ (C_3_ × C_4_), and C_3_+C_4_ and C_4_ (C_3_+C_4_ × C_4_) accessions of *A. semialata* and analyse their phenotype to test the hypotheses that components of the C_4_ trait are additive, and that hybrids rank between their non‐C_4_ and C_4_ parents in terms of photosynthetic performance. Our study confirms that crosses between photosynthetic types in *A. semialata* are viable in experimental conditions, and shows that the C_4_ metabolism is disrupted in the hybrids despite the significant upregulation of anatomical and biochemical components of the C_4_ trait.

## 2 MATERIALS AND METHODS

### 2.1 Plant material and growth conditions

Crosses between accessions of *Alloteropsis semialata* (R. Br.) Hitchc. (Poaceae) from distinct geographical origins were generated from parental plants collected in the wild as seeds or cuttings (Table S1) and grown in greenhouse conditions at the Arthur Willis Environment Centre, University of Sheffield (UK). Initial attempts of controlled cross‐pollination using pollination bags to isolate hand‐pollinated inflorescences were unsuccessful. For this reason, we adopted a noncontrolled pollination strategy, in which plants were allowed to receive pollen from any other plant in the greenhouse, with subsequent determination of the pollen parent via genotyping (see below). The resulting seeds were collected while still attached to the mother plant, and subsequently germinated on Petri dishes before being potted. Here, a total of 31 seedlings from seven mother plants (two C_3_, two C_3_+C_4_ and three C_4_) were obtained. Subsequent genotyping of the seedlings identified multiple F1 hybrids between parents with distinct photosynthetic types, including 14 C_3_ × C_4_ and 6 C_3_+C_4_ × C_4_ individuals (Table S1). The remaining seedlings consisted of 10 C_4_ × C_4_ crosses, and an additional C_3_+C_4_ plant resulting from self‐pollination (Table S2). Five C_3_ × C_4_ individuals grew to maturity and were sampled for DNA analyses but died before being phenotyped, and another two died before being sampled for RNA sequencing and leaf gas‐exchange analyses (Table S1). For the phenotypic analyses, we included the mother plants, the known and possible pollen parents (i.e., similar C_4_ accessions that could not be distinguished by the genotyping analyses; see Table S1), and seven additional accessions of *A. semialata* (three C_3_, two C_3_+C_4_ and two C_4_) that were growing alongside them were added to increase the phenotypic diversity within photosynthetic types (Table S1). All plants were grown in 11‐L, free‐draining pots containing a 2:1 mix of M3 compost (Levington) and perlite (Sinclair) under well‐watered conditions, and were fertilized once every 3 months with an NK fertilizer 16‐0‐5 containing Iron (Evergreen Extreme Green Lawn Food). Plants were grown on a 12‐h day/night cycle, with metal halide lamps providing supplementary light (additional 200 μmol m^−2^ s^−1^ at bench level), 25/20°C day/night temperature, ambient CO_2_ concentration and relative humidity between 30% and 60%.

### 2.2 Genotyping

We used three genetic markers to determine the genetic lineage of the pollen parent of each plant. These markers correspond to regions of the nuclear genome (~500 bp long) with sufficient variation to distinguish between different photosynthetic types and geographical groups, and were selected from a set of markers previously assembled to determine the origin of allopolyploids in *A. semialata* (Bianconi et al., 2020). Custom primers were designed to PCR amplify the target genes (Table S3). Genomic DNA (gDNA) of the putative crosses was isolated from fresh leaves using the DNeasy Plant Mini Kit (Qiagen). PCR reactions contained ca. 10–40 ng of gDNA template, 5 µl of 5× GoTaq Flexi reaction buffer (Promega), 2 mM of MgCl_2_, 0.08 mM of dNTPs, 0.2 µM of each primer and 0.5 unit of GoTaq polymerase (Promega) in a total volume of 25 µl. The PCR mixtures were initially incubated in a thermocycler for 2 min at 94°C followed by 35 cycles consisting of 30 s at 94°C (denaturation), 1 min at 48°C (annealing) and 1 min at 72°C (elongation). Amplicons were cleaned using Exo‐SAP‐IT (Affymetrix), and Sanger‐sequenced at the Core Genomic Facility at the University of Sheffield. Sequencing chromatograms were individually inspected for heterozygous sites that are polymorphic among genetic groups corresponding to distinct photosynthetic types, since the presence of such heterozygous sites indicates an F1 hybrid between photosynthetic types. Because the maternal origin was known in advance (the maternal plant was the one on which the seed was collected), the information from the alternative bases was used to narrow down the pollen parent to a genetic lineage (Table S2). The hybrid origin of the plants was subsequently confirmed using RNAseq data (see below).

### 2.3 Stable carbon isotopes

Carbon isotope composition was determined from the central portion of fully expanded leaf blades. Samples were dried in silica gel, and subsequently ground to a fine powder using a TissueLyzer II (Qiagen). Carbon isotope analysis was conducted on 1–2 mg of leaf sample using an ANCA GSL preparation module coupled to a Sercon 20‐20 stable isotope ratio mass spectrometer (PDZ Europa). Carbon isotopic ratios (δ^13^C, in ‰) were reported relative to the standard Pee Dee Belemnite (PDB). Values of δ^13^C higher than −16‰ indicate that the plants grew using C_4_ photosynthesis (O'Leary, 1988; Stata et al., 2019). δ^13^C values of additional accessions of *A. semialata* that were previously phenotyped and identified as C_3_, C_3_+C_4_ or C_4_ (Dunning et al., 2017, 2019a; Lundgren et al., 2016, 2019) were retrieved from Lundgren et al. (2015, 2016).

### 2.4 Leaf anatomy

Leaf cross sections were obtained from the central portion of fully expanded leaf blades. Fresh samples were initially dehydrated in an ethanol series from 70% to 100% EtOH, and resin‐infiltrated with Technovit 7100 (Heraeus Kulzer GmbH) following the manufacturer's instructions. Cross sections 7–10‐μm‐thick were obtained using a microtome (Leica RM 2245; Leica Biosystems Nussloch GmbH), and stained with Toluidine Blue O (Sigma‐Aldrich). An Olympus BX51 microscope coupled to an Olympus DP71 camera (Olympus Corporation) was used to photograph the cross sections. Leaf anatomical traits were measured using ImageJ v.1.51q (Schneider et al., 2012) in one cross section per individual. Measurements were taken following Lundgren et al. (2019) within segments, where the segment was defined as the area between two consecutive secondary veins (i.e., veins with large metaxylem vessels; see Figure S1). Segments that were adjacent to the midvein or to the lateral margins of the cross section were avoided to maintain consistency between samples. The variables measured here included leaf thickness (at the leftmost secondary vein in the segment), the number of minor veins (4th and 5th order veins), vein density, distance between edges of consecutive bundle sheaths (BSD), width of inner (IS) and outer sheath (OS), and the areas of epidermis (including bulliform cells, Epd), mesophyll (M), extraxylary fibres (Fb), bundle sheath (separately for IS and OS) and vascular tissue (V; see Figure S1 for an illustrated key for each variable). Areas were expressed relative to the total area of the segment before analysis, except IS and OS, which were expressed relative to the mesophyll area (IS/M and OS/M). A principal component analysis (PCA) was performed on these 12 leaf anatomical variables using the function *prcomp* in R v. 3.6.3 (R Core Team, 2020).

### 2.5 Leaf transcriptome

Leaf mRNA was isolated and sequenced as previously described (Dunning et al., 2019a). In short, the distal halves of fully expanded leaves were sampled in the middle of the light period, flash‐frozen in liquid N_2_ and kept at −80°C. All samples were collected on the same day. Total RNA was extracted using the RNeasy Plant Mini Kit (Qiagen) with an on‐column DNA digestion step (RNase‐Free DNase Set; Qiagen). A total of 20 RNA‐Seq libraries (one per individual) were prepared with the TruSeq RNA Library Preparation Kit v2 (Illumina) using 0.5 μg of starting RNA and aiming at a median insert size of ~155 bp (standard fragmentation protocol). Libraries were paired‐end sequenced (read length = 100 bp) on 1/24 of a single lane of an Illumina HiSeq 2500 flow cell in rapid mode (with four additional samples from an unrelated project) at the Sheffield Diagnostic Genetics Service. Raw sequence reads were filtered to remove adaptor contamination and low‐quality reads (i.e., <80% of bases with Phred score >20) using NGSQCToolkit v. 2.3.3 (Patel & Jain, 2012). Reads were further trimmed from the 3′ end to remove bases with Phred score <20. The quality of the filtered data was assessed using FastQC v. 0.11.9 (Andrews, 2010).

Transcript abundance was quantified by mapping the filtered reads to a reference data set consisting of coding sequences from multiple transcriptomes of *A. semialata* retrieved from Dunning et al. (2017) and modified by Bianconi et al. (2018). In short, this data set consists of 5540 groups of homologous genes that are common to the Panicoideae grasses (the subfamily that includes *Alloteropsis*), each group containing all paralogs detected in the transcriptomes of *A. semialata* (total of 12 234 groups of co‐orthologs). We used this data set as it includes manually curated sets of co‐orthologs of 23 gene families known to have a function in the C_4_ biochemistry (Bianconi et al., 2018). This curated gene set increases the read mapping accuracy when paralogs exist with a high sequence similarity. Furthermore, this data set includes the sequences of paralogs of core C_4_ genes that are absent from the reference genome of *A. semialata*, such as four different genes for the enzyme phosphoenolpyruvate carboxylase (PEPC) known to be highly expressed in other *A. semialata* accessions (Dunning et al., 2017, 2019a). Filtered reads were mapped to the reference data set using Bowtie2 v. 2.3.5 (Langmead & Salzberg, 2012) with default parameters. Read counts are reported here in reads per million mapped reads (RPM). To visualize the diversity among samples, a PCA on the global patterns of gene expression was conducted using the function *prcomp* in R after log2‐transforming the RPM values. Only genes with transcript abundance >10 RPM in at least five samples were used for the PCA. To test whether the global patterns of gene expression were consistent when the whole gene set of *A. semialata* was considered, we repeated the analysis using the chromosome‐level genome assembly of this species (Dunning et al., 2019b) as reference for read mapping. Finally, to verify whether the expression level differences between photosynthetic types were consistent with previous reports for *A. semialata*, we quantified transcript abundance (using the co‐orthologous gene set as reference) for 20 additional accessions (four C_3_, six C_3_+C_4_ and 10 C_4_) retrieved from two previous RNAseq studies (Dunning et al., 2017, 2019a).

To explore the global differences in gene expression between hybrids and the parental types, we performed a differential expression analysis on the data set that was generated using the complete genome of *A. semialata* as reference for read mapping. Here, due to an insufficient number of replicates for C_3_ and C_3_+C_4_ plants, we restricted our analyses to the comparisons between the two hybrid types, and between each of these and the C_4_ group. We then investigated the main metabolic functions associated with the set of differentially expressed genes from each comparison (see full description of the global RNAseq analyses on the Supporting Information Methods).

Finally, to confirm the paternity of the putative hybrids, we genotyped the plants using the RNAseq data set mapped to the reference genome of *A. semialata*. Here we also included individuals of *A. semialata* from the RNAseq studies of Dunning et al. (2017, 2019a) to increase the genetic diversity of the data set. Read alignment files were filtered to remove duplicates using Picard Tools v. 1.102 (https://broadinstitute.github.io/picard/), and variants were called on all individuals combined using BCFtools v. 1.9 (Li et al., 2009). Variant sites were filtered using VCFtools v. 0.1.17 (Li et al., 2009) to remove low‐quality genotype calls (i.e., quality score <30 and read depth <3), indels, and sites with more than 10% missing data, which resulted in an initial set of 521 929 single‐nucleotide polymorphisms (SNPs). BCFtools was then used to retain only multiallelic sites in which all hybrids were heterozygous and all potential parents were homozygous, which resulted in 15 406 SNPs that were then used for the genotyping tests. First, to determine the type of cross (i.e., whether C_3_ × C_4_ or C_3_+C_4_ × C_4_), we identified SNPs that were common to all individuals of the same photosynthetic type and exclusive to them. Sites with more than 25% missing data within each photosynthetic group were removed, and a total of 114 SNPs were retained (Table S4). We then tested, for each SNP, whether hybrid individuals had one C_4_ allele and one allele that was either C_3_ or C_3_+C_4_, according to the type of cross (Table S4). With the confirmation of the type of cross, we then narrowed down the genetic lineage of the pollen parent (as the mother is already known). Only one individual representing each genetic lineage was retained for this analysis (Table S5). First, we used the 15 406 SNP set to identify all singletons of each potential pollen parent, which in this case were defined as homozygous genotypes that were unique to a single individual among all individuals with the same photosynthetic type. A total of 7779 singletons were identified (minimum of 287 per potential pollen parent; Table S5). We then verified, for each singleton, which hybrid individuals carried that same allele, and counted the number of positive matches (Table S5). All genotyping analyses were conducted using R (scripts are provided as Supplementary Files).

### 2.6 Leaf gas‐exchanges

The photosynthetic response to intercellular CO_2_ concentration (*A*/*Ci* curve) was measured using two portable gas‐exchange systems Li‐6400XT (Li‐Cor). *A*/*Ci* curves were measured within the first 6 h of the photoperiod on the widest fully expanded leaf at a block temperature (*T*_block_) of 25°C, flow rate = 300 μmol s^−1^, photosynthetic photon flux density (*PPFD*) = 1500 μmol m^−2^ s^−1^, and were started after both net CO_2_ uptake (*A*) and stomatal conductance (*g*_S_) reached steady state at reference CO_2_ = 400 μmol mol^−1^. Reference CO_2_ was then changed in a stepwise manner to 250, 150, 120, 100, 85, 70, 50, 35, 400, 600, 800, 1000 and 1200 μmol mol^−1^. Readings were automatically logged after 2–3 min of leaf acclimation to each CO_2_ level. The CO_2_ compensation point (CCP) and maximum carboxylation efficiency (CE) were calculated following Bellasio et al. (2016). In this approach, the CO_2_‐dependence of *A* is described by an empirical nonrectangular hyperbola, and allows for the estimation of parameters irrespective of the photosynthetic physiology, which is therefore a suitable approach for hybrids (Bellasio et al., 2016). Water‐use efficiency (WUE) was calculated as the ratio between *A* and *g*_S_ at steady state at reference CO_2_ = 400 μmol mol^−1^.

Stomatal density on the abaxial side of the leaves was quantified using leaf impressions. Dental resin (ImpressPlus Wash Light Body, Perfection Plus Ltd.) was applied to the central portion of fully expanded leaves, and clear nail varnish was applied to the set resin impression. Images were captured from the nail varnish impressions at 20× using an Olympus BX51 microscope coupled to an Olympus DP71 camera, and stomatal density was quantified on 0.38 mm² fields using ImageJ.

## 3 RESULTS

### 3.1 Carbon isotopic ratios are intermediate between the parents

**Figure 1:**
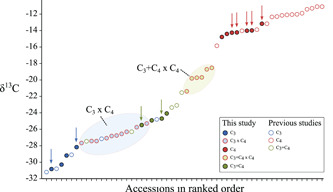
Distribution of carbon isotopic ratios (δ^13^C) in *Alloteropsis semialata*. δ^13^C values of accessions of *A. semialata* that are not part of this study were retrieved from Bianconi et al. (2020). Blue, green and red arrows indicate δ^13^C values of C_3_, C_3_+C_4_ and C_4_ known or potential parents of the hybrids analysed in this study [Color figure can be viewed at wileyonlinelibrary.com]

Carbon isotopic ratios (δ^13^C) of all hybrids between C_4_ and non‐C_4_ accessions were below the range of C_4_ values in *A. semialata* (Figure 1 and Table S1). However, δ^13^C was consistently higher in the hybrids than in their non‐C_4_ parents, with average increases of 2.8‰ in C_3_ × C_4_ and 5.4‰ in C_3_+C_4_ × C_4_ hybrids relative to their C_3_ and C_3_+C_4_ parents, respectively. The δ^13^C ranged between −27.7 and −25.3‰ in C_3_ × C_4_ and −21.4 and −18.5‰ in C_3_+C_4_ × C_4_ hybrids.

### 3.2 Leaf anatomy is additive in the hybrids

**Figure 2:**
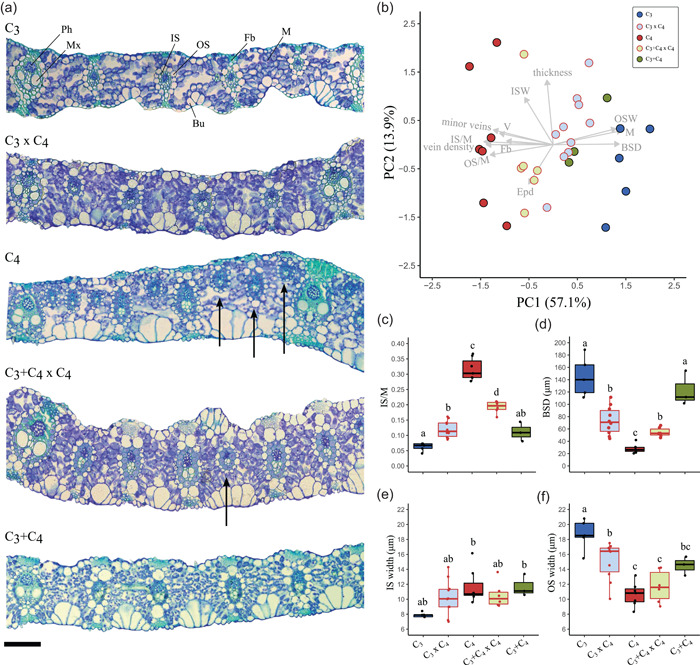
Leaf anatomy of F1 hybrids and the parental photosynthetic types in *Alloteropsis semialata*. (a) Representative cross sections of C_3_ (RSA9), C_3_ × C_4_ (H08), C_4_ (TPE1‐10), C_3_+C_4_ × C_4_ (H11) and C_3_+C_4_ (TAN1602‐03c) accessions. Arrows indicate minor veins (i.e., fourth‐ and fifth‐order veins). Scale bar = 100 µm. (b) Principal component analysis of selected leaf anatomical variables. (c) Proportion of inner sheath to mesophyll area (IS/M). (d) Distance between consecutive bundle sheaths (BSD). (e) Mean inner sheath cell width. (f) Mean outer sheath cell width. For (c–f), different lower‐case letters indicate statistical differences between groups (ANOVA, *p* < 0.05 post hoc Tukey HSD; *n* ≥ 4). Bu, bulliform cells; BSD, distance between consecutive bundle sheaths; Epd, proportion of epidermis area; Fb, extraxylary fibres (=sclerenchyma girder); IS, inner bundle sheath (=mestome sheath); ISW, IS width; M, mesophyll; Mx, metaxylem; OS, outer bundle sheath; OSW, OS width; Ph, phloem; V, proportion of vascular tissue area

C_4_ and non‐C_4_ leaves in *A. semialata* are mainly distinguished by the presence of minor veins in C_4_ accessions (Lundgren et al., 2019). Here, minor veins were observed in all hybrids, except two C_3_ × C_4_ individuals (Table S6). The presence of chloroplasts in the inner sheath of all hybrids is suggested by the strong and consistent staining patterns in these cells, which are similar to the patterns observed in C_4_ and C_3_+C_4_ *A. semialata*, but not in C_3_ accessions, where the staining is very weak (Figure 2a). The first component of a PCA on 12 quantitative anatomical traits explains 57.1% of the total variation and separates the accessions by photosynthetic type (Figure 2b). C_4_ individuals are associated with negative values of the first component, which correspond to increased vein density (including minor veins) and increased abundance of bundle sheath tissue (IS and OS) relative to the mesophyll (Figure 2b). C_3_+C_4_ and C_3_ accessions partially overlap in the PCA space, and are associated with positive values of the first component. Hybrid individuals are intermediate between their respective parents along this first component. The second principal component explains 13.9% of the variance, and is correlated with leaf thickness (*r* = 0.88, *p* < 0.001), width of inner bundle sheath cells (ISW; *r* = 0.64, *p* < 0.001) and epidermis area (Epd; *r* = −0.54, *p* < 0.001), but these variables do not differentiate photosynthetic or cross types. The proportion of inner bundle sheath relative to mesophyll (IS/M) was on average doubled in C_3_ × C_4_ hybrids compared to C_3_ accessions, and was within the range of C_3_+C_4_, but the values were still 62% lower than the C_4_ average (Figure 2c). The distance between consecutive bundle sheaths (BSD) in C_3_ × C_4_ and C_3_+C_4_ × C_4_ hybrids was on average 49% and 55% smaller than in C_3_ and C_3_+C_4_ plants, respectively (Figure 2d). The mean width of IS cells was similar between C_3_+C_4_ and C_4_ accessions, and values were on average 48% higher than in C_3_, but their differences among photosynthetic types or between hybrids and the respective parental types were not significant (Figure 2e). OS cells were however significantly smaller in C_4_ than in C_3_ accessions, with hybrids having intermediate values that were not significantly different from those of C_3_+C_4_ accessions (Figure 2f). In the hybrids, the variation in BSD was largely explained by vein density (*R* ^2^ = 0.92, *p* < 0.001), with no significant effects of OS or IS widths. The IS/M ratio, on the other hand, was not significantly associated with vein density, but with both OS and IS width (*R* ^2^ = 0.75, *p* < 0.001). Overall, C_3_ × C_4_ hybrids were not significantly different from C_3_+C_4_ in relation to the quantitative anatomical traits analysed here, except for BSD, which was significantly reduced in C_3_ × C_4_ as a result of the presence of some minor veins in most accessions. In C_3_+C_4_ × C_4_ hybrids, a quantitatively distinct phenotype was generated, with trait values ranking between C_3_+C_4_ and C_4_ plants. Overall, our results show that leaf anatomical traits in *A. semialata* are mostly inherited in an additive manner.

### 3.3 Gene expression

**Figure 3:**
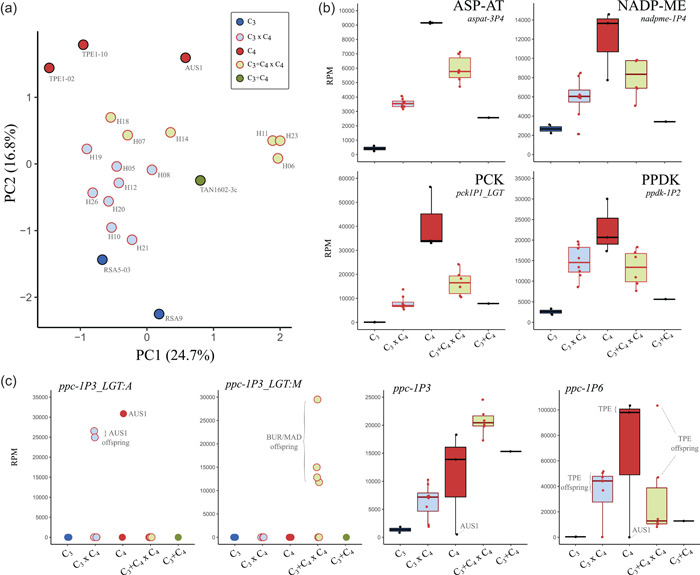
Leaf gene expression of F1 hybrids and the parental photosynthetic types in *Alloteropsis semialata*. (a) Principal component analysis on 7482 genes. (b) Transcript abundance in reads per million mapped reads (RPM) of selected genes encoding core C_4_ enzymes: aspartate aminotransferase (ASP‐AT, gene *aspat‐3P4*), NADP‐dependent malic enzyme (NADP‐ME, gene *nadpme‐1P4*), phosphoenolpyruvate carboxykinase (PCK, gene *pck1P1_LGT*), pyruvate phosphate dikinase (PPDK, gene *ppdk‐1P2*). (c) Transcript abundance of selected phosphoenolpyruvate carboxylase (PEPC) genes [Color figure can be viewed at wileyonlinelibrary.com]

Global gene expression patterns were assessed via a PCA on 7482 genes (Figure 3a). The first principal component mostly separated three sister C_3_+C_4_ × C_4_ plants (H06, H11, and H23) from the other individuals, and accounted for 24.7% of the variation. Photosynthetic types were clearly separated by the second principal component, which accounted for 16.8% of the variation and placed the hybrids as intermediate to the photosynthetic types of their parents. Similar clustering patterns were observed when transcript abundance was quantified for the complete gene set extracted from the genome of *A. semialata* (Figure S2).

Transcript abundance of five genes encoding key enzymes for the C_4_ biochemistry, and which are known to be upregulated in C_4_ plants of *A. semialata* (Dunning et al., 2019a), were analysed in detail (Figure 3b,c and Table S7). The patterns of transcript abundance of C_3_, C_3_+C_4_ and C_4_ plants are highly similar to those reported by Dunning et al. (2017, 2019a), where a larger sample size was used (Figure S3). Transcript abundance of the genes encoding the C_4_‐specific forms of the enzymes aspartate aminotransferase (ASP‐AT, gene *aspat‐3P4*), NADP‐malic enzyme (NADP‐ME, gene *nadpme‐1P4*) and pyruvate phosphate dikinase (PPDK, gene *ppdk‐1P2*) were 4‐ to 17‐fold higher in C_4_ than in C_3_ plants, with the transcript abundances of the C_3_+C_4_ plant slightly above those of C_3_ plants (Figure 3b). In each case, the transcript abundance of the hybrids was intermediate to the abundance of the photosynthetic types of the parents, showing that these expression patterns are mostly additive. C_3_ accessions of *A. semialata* lack the gene copy encoding phosphoenolpyruvate carboxykinase (PCK) that is highly expressed in C_4_ and C_3_+C_4_ accessions (*pck‐1P1_LGT*; Olofsson et al., 2016); the transcript abundance of this gene reaches 20% of the mean C_4_ values in C_3_ × C_4_ hybrids, and 40% in C_3_+C_4_ × C_4_.

Out of the nine genes encoding the key C_4_ enzyme phosphoenolpyruvate carboxylase (PEPC) that have been identified in *A. semialata* accessions (Dunning et al., 2017), only five were reported to be highly expressed in leaves of some C_4_ accessions (Christin et al., 2012; Dunning et al., 2017, 2019a). One of these genes, *ppc‐1P3_LGT:A* is only present in the C_4_ lineage from Oceania, which is represented here by an Australian accession (AUS1) that is the pollen parent of two C_3_ × C_4_ hybrids (H19 and H21; Table S5); both plants have expression levels of this gene that are similar to the Australian parent (Figure 3c). Only one of the other two laterally acquired PEPC copies is present in the plants analysed here (*ppc‐1P3_LGT:M*), and it is highly expressed (>10 000 RPM) in four C_3_+C_4_ × C_4_ individuals that are offspring from the C_4_ accessions from Burkina Faso or Madagascar (H06, H11, H14 and H23; Table S5 and Figure 3c), which have been previously shown to be part of the only C_4_ lineage to carry this gene (Olofsson et al., 2016). The expression levels of *ppc‐1P3* are similar across C_3_+C_4_ and C_4_ accessions, and are on average 10‐fold higher than in C_3_ accessions (except the C_4_ AUS1, which has a nonfunctional copy of this gene; Olofsson et al., 2016); in the C_3_+C_4_ × C_4_ hybrids, however, the transcript abundance of this gene is on average 42% higher than the median of C_3_+C_4_ and C_4_ accessions (Figure 3c). Finally, the gene *ppc‐1P6* reaches high expression levels in C_4_ accessions (except in AUS1, where it is absent; Dunning et al., 2019b), but is disproportionally upregulated in the C_4_ Taiwanese lineage (TPE; Dunning et al., 2017), where it reaches levels that are up to 100‐fold higher than in C_3_ plants, and at least twice higher than the values in other C_4_ and C_3_+C_4_ accessions. Here, the *ppc‐1P6* copy reaches high levels in the C_3_ × C_4_ and C_3_+C_4_ × C_4_ offspring of TPE, mostly ranging from 10 000 to 60 000 RPM, except for one C_3_+C_4_ × C_4_ which has a transcript abundance similar to TPE (~100 000 RPM).

We examined transcript abundance of selected protein‐coding genes with a role in photorespiratory metabolism. We observed reduced transcript levels of the genes encoding the peroxisomal enzymes flavin mononucleotide‐dependent glycolate oxidase (GOX, gene *glo‐1P2*) and glutamate:glyoxylate aminotransferase (GGT, gene *ggat‐1P6*) in C_3_+C_4_ × C_4_ hybrids relative to C_3_ and C_3_+C_4_ accessions, with average values that are similar to or below those of C_4_ accessions (Figure S4). The genes encoding the proteins P‐, T‐, L‐ and H‐ that compose the mitochondrial multienzyme system glycine decarboxylase (GDC, genes *gldp‐1P1*, *gcvt‐1P1*, *lpd‐1P2* and *gdh‐2P2*, respectively) had on average 63% lower transcript levels in C_4_ than in C_3_ accessions; in C_3_ × C_4_ and C_3_+C_4_ × C_4_ hybrids, transcript levels of these four genes were on average 12% and 33% lower than in C_3_, respectively, suggesting additivity (Figure S4 and Table S8). Multiple genes encoding a serine hydroxymethyltransferase (SHMT) are found in the genome of *A. semialata* (genes *shm‐1P1*, *shm‐2P2*, *shm‐3P3* and *shm‐3P4*; Dunning et al., 2019a), but none had patterns of transcript abundance similar to those observed for GDC; in fact, one of the genes (*shm‐3P3*) had higher average transcript abundance in C_4_ than in C_3_ accessions. The chloroplast genes glycerate 3‐kinase (GLYK, gene *glyk‐1P1*) and 2‐phosphoglycolate (2‐PG) phosphatase (PGLP, gene *pglp‐2P2*) had overlapping transcript abundance ranges across hybrid and photosynthetic types. Finally, a gene encoding a GOLDEN2‐LIKE transcription factor that has been previously demonstrated to be involved in the redistribution of organelles among cell types (Wang et al., 2017) is expressed at higher levels in C_4_ than in C_3_ and C_3_+C_4_ accessions (Figure S4L and Table S8). This gene is at intermediate levels in the C_3_ × C_4_ hybrids, but largely variable among C_3_+C_4_ × C_4_ individuals.

We identified genes differentially expressed (DE) between hybrid types, and between these and C_4_ accessions (Table S9). We found a total of 4104 genes differentially expressed between C_3_ × C_4_ and C_3_+C_4_ × C_4_ hybrids (adjusted *p* value <0.05), out of which 21 were related to C_4_ photosynthesis and 4 to photorespiration (Table S9). This complete DE gene set is reduced to 206 when only higher count genes (base mean >500) and with larger differences (log2fold change >1) are considered (Table S9). Among this reduced gene set, we found seven C_4_‐related genes, but none related to photorespiration (Figure S5 and Table S9). The individual comparisons between C_3_ × C_4_ or C_3_+C_4_ × C_4_ and C_4_ accessions identified 1925 and 2727 significant DE genes, respectively. These numbers are reduced to 110 and 181 when the same filters described above are applied (Table S9). In this reduced set, we found 13 C_4_‐related genes that were differentially expressed between hybrids and C_4_ accessions, 3 of these common to both C_3_ × C_4_ vs. C_4_ and C_3_+C_4_ × C_4_ vs. C_4_ comparisons, namely PCK (*pck‐1P1_LGT*), NADP‐malate dehydrogenase (NADP‐MDH, gene *nadpmdh‐1P2*) and soluble inorganic pyrophosphatase (PPA, gene *ppa‐2P1*). While *nadpmdh‐1P2* was downregulated in the C_4_ group, the two others had increased transcript abundance in C_4_ relative to both hybrid types (Figure S5). Finally, we found four genes related to photorespiration in the DE gene set between C_3_ × C_4_ and C_4_ accessions, three of which were upregulated in C_3_ × C_4_, namely the genes encoding the GDC proteins T‐ (*gcvt‐1P1*), P‐ (*gldp1P*) and L‐ (*lpd‐1P2*; Figure S5). The remaining gene, which encode the enzyme SHMT (*shm‐3P3*) was downregulated in both C_3_ × C_4_ and C_3_+C_4_ × C_4_ hybrids relative to C_4_ accessions. Gene ontology (GO) analyses did not detect any significantly enriched cellular processes for any of the full DE gene sets. However, the reduced DE gene set from the comparison between hybrid types was significantly enriched with genes related to the photosystems I and II, oxidoreductase activity, and 1,3‐beta‐d‐glucan synthesis (Table S10). In the reduced gene set from comparisons between each of the hybrid types and the C_4_ type, GO analyses indicated a significant enrichment for genes associated with C_4_ photosynthesis (particularly malate metabolism), transmembrane transport, and photosystems I and II (Table S10).

Overall, the analyses of expression profiles show that the inheritance of expression levels of C_4_ genes and those involved in the photorespiratory pathway is largely additive. In almost all cases, the transcript abundance of the hybrids ranks between those of the parental lineages.

### 3.4 Evidence of reduced photosynthetic performance in the hybrids

**Figure 4:**
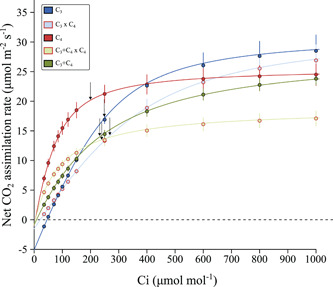
Response of net CO_2_ assimilation rate (*A*) to intercellular CO_2_ (*Ci*) of F1 hybrids and the parental photosynthetic types in *Alloteropsis semialata*. Data points are predicted values from an empirical nonrectangular hyperbola model (Bellasio et al., 2016) based on the mean parameter estimates per individual within each cross/photosynthetic type. Error bars show standard errors for the predicted values (*n* ≥ 3). Arrows indicate *Ci* values at reference CO_2_ = 400 µmol mol^−1^ for each cross/photosynthetic type [Color figure can be viewed at wileyonlinelibrary.com]

**Figure 5:**
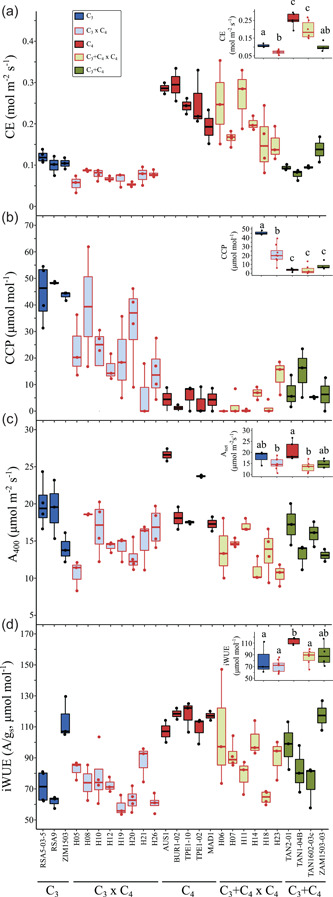
Photosynthetic performance of F1 hybrids and the parental photosynthetic types in *Alloteropsis semialata*. (a) Maximum carboxylation efficiency (CE), (b) CO_2_ compensation point (CCP), and steady‐state (c) net photosynthetic rate (*A*_400_), and (d) intrinsic water use efficiency (iWUE, *A*/*g*_s_) at reference CO_2_ = 400 µmol mol^−1^. Different lower‐case letters indicate statistical differences between groups (ANOVA, *p* < 0.05 post hoc Tukey HSD; *n* ≥ 4) [Color figure can be viewed at wileyonlinelibrary.com]

Photosynthetic performance was assessed from CO_2_ response curves (*A*/*Ci*) and steady‐state measurements at ambient CO_2_ (Figure S6; Tables S11 and S12). Photosynthetic rates in the hybrids ranked between the parents at low Ci levels, but the differences between hybrids and parents were progressively reduced as CO_2_ levels increased, until they were equal to their non‐C_4_ parents at Ci of 100 in C_3_ × C_4_, and 250 µmol mol^−1^ in C_3_+C_4_ × C_4_ hybrids (Figure 4). The C_3_ and C_3_+C_4_ accessions had on average similar values of maximum CE, and these were 57% lower than in C_4_ accessions (Figure 5a and Table S11). Despite the larger variation among individuals, C_3_+C_4_ × C_4_ hybrids had CE values that were on average 83% and 96% higher than in C_3_ and C_3_+C_4_ accessions, respectively, and these were not significantly different from the average values of C_4_ accessions. In C_3_ × C_4_ hybrids, however, CE was consistently lower than in C_3_ accessions, and reached the lowest mean values of all photosynthetic types/crosses. The CCP ranged between 43 and 48 µmol mol^−1^ in C_3_ accessions, and between 5 and 15 µmol mol^−1^ in C_3_+C_4_ individuals (Figure 5b). All C_4_ had CCP below 10 µmol mol^−1^. In the hybrids, CCP was below 13 µmol mol^−1^ in C_3_+C_4_ × C_4_ individuals, and ranged between 6 and 40 µmol mol^−1^ in C_3_ × C_4_ hybrids. CCP estimates varied substantially among *A*/*Ci* curves in C_3_ × C_4_ individuals (Figure 5b), but mean values were 52% lower than in C_3_ plants.

The steady‐state net CO_2_ assimilation rate at ambient CO_2_ (400 µmol mol^−1^; *A*_400_) was highest in two C_4_ accessions, but the differences between C_3_, C_3_+C_4_ and C_4_ plants were not significant (Figure 5c). C_3_+C_4_ × C_4_ hybrids had the lowest *A*_400_, but mean values were not significantly different from C_3_ × C_4_, C_3_ and C_3_+C_4_ accessions. Stomatal conductance (*g*_s_) had the highest median values in C_3_ accessions, but the differences between groups were not significant (Figure S7A). C_3_ accessions had on average 50% higher stomatal density than C_3_+C_4_ and C_4_ accessions and their hybrids (Figure S7B). We found a positive correlation between *g*_s_ and stomatal density (*R*² = 0.31, *p* < 0.01), but only after excluding the outlier C_3_ accession ZIM1503, which had the highest stomatal density, but one of the lowest *g*_s_. The correlation between stomatal density and vein density was only marginally significant (*R*² = 0.01, *p* = 0.06). The intrinsic water use efficiency (iWUE) at ambient CO_2_ was on average 38% higher in C_4_ than in other accessions, although a few C_3_, C_3_+C_4_ and C_3_+C_4_ × C_4_ individuals also showed C_4_‐like values (Figure 5d). The lowest iWUE values were observed in C_3_ and C_3_  × C_4_ individuals, but the means were not significantly different from C_3_+C_4_ and C_3_+C_4_ × C_4_ hybrids. To investigate whether changes in leaf temperature (*T*_leaf_) could explain the observed patterns, we inspected the variation in *T*_leaf_ during the *A*/*Ci* curves. Although *T*_leaf_ values up to 3°C above the median (26.3°C) were observed in a few accessions, 81% of all data points were collected within a 2°C interval (Figure S8). There were no significant differences between groups when all points were considered, nor when only above‐ or below‐ambient CO_2_ levels were analysed (Figure S8). Similar patterns among cross/photosynthetic types were observed in all photosynthetic parameters after removing individuals for which the median *T*_leaf_ was 1°C above the median of all curves (Figure S9).

Overall, these results show that the physiological characters are not consistently additive in the hybrids, with some individuals performing below their two parents for several traits.

## 4 DISCUSSION

### 4.1 Partial contribution of the C_4_ cycle to carbon fixation in hybrids

In this study, we analyse the phenotypes of hybrids between individuals of the grass *A. semialata* with different photosynthetic types. The C_3_ × C_4_ quantitatively resemble the naturally occurring C_3_+C_4_ in most anatomical and gene expression traits (Figures 2 and 3), as previously noticed in other taxa (Kadereit et al., 2017). They however differ qualitatively, and minor veins and high expression of some C_4_‐related genes are especially restricted to C_3_ × C_4_ individuals (Figure 3 and Table S6). These findings therefore provide additional support for the hypothesis that naturally occurring C_3_+C_4_ in *A. semialata* do not result from recent crosses between photosynthetic types (Lundgren et al., 2015, 2016), although genome analyses support a role for introgression in their distant history (Bianconi et al., 2020).

Across all crosses, we show that hybrids between C_4_ and non‐C_4_ individuals of the grass *A. semialata* do not exhibit a full C_4_ physiology, as indicated by their δ^13^C, which are clearly outside the range of C_4_ values in the species (Figure 1). This indicates that a significant fraction of CO_2_ assimilation in these hybrids occurs directly via Rubisco in the mesophyll, which implies that the genetic contribution of the C_4_ parent was insufficient to (1) completely suppress Rubisco expression and/or activity in the mesophyll cells and/or (2) create an effective C_4_ cycle. However, the CCP of the C_3_ × C_4_ hybrids is substantially reduced compared to the C_3_ parents (Figure 5b). While this change could theoretically result from an increase of the photorespiratory shuttle observed in many C_3_–C_4_ plants (called ‘C_2_' plants; Keerberg et al., 2014; Khoshravesh et al., 2016), the expression of photorespiration‐related genes is rather unchanged or decreased in our hybrids compared to the C_4_ parents. Changes in CCP are therefore more likely linked to an increase in the C_4_ activity, which is further supported by significantly higher δ^13^C values compared to their non‐C_4_ parents (Figure 1). Our results therefore suggest that, in most hybrids, a fraction of the CO_2_ that enters the leaf is fixed by PEPC in the mesophyll and follows the C_4_ route to be refixed by Rubisco in the inner bundle sheath. Furthermore, given the C_4_‐like CCP and CE values, and δ^13^C above −20‰ (in most cases), the degree of C_4_ activity is higher in C_3_+C_4_ × C_4_ than in C_3_ × C_4_ hybrids. Our findings therefore recapitulate what has been shown for interspecific hybrids between photosynthetic types in several eudicot and monocot genera (Araus et al., 1990; Björkman et al., 1969; Brown & Bouton, 1993; Brown et al., 1985; Byrd et al., 1992; Oakley et al., 2014; Sultmanis, 2018). The causes for this only partial contribution of the C_4_ pathway to the overall carbon assimilation in the hybrids are potentially the same in all these cases, and are probably related to the insufficient expression of components of C_4_ metabolism (Brown & Bouton, 1993).

Two interrelated leaf anatomical traits were significantly different from the C_4_ individuals in both C_3_ × C_4_ and C_3_+C_4_ × C_4_ hybrids, namely the proportion of inner sheath to mesophyll tissue (IS/M) and the distance between consecutive bundle sheaths (BSD). These traits are mostly determined by the presence of minor veins in C_4_ leaves, which has been previously shown to be the major anatomical difference between C_4_ and non‐C_4_ *A. semialata* (Lundgren et al., 2019). Here, significant changes towards the C_4_ phenotype in IS/M and BSD were observed in the hybrids, but realized values were still far from those observed in C_4_ accessions (Figure 2c,d). This is particularly clear for IS/M, where values were below the mean of both parents in the two hybrid types. While minor veins are observed in the hybrids, their number per segment remains below that observed in C_4_ accessions (Table S6). This indicates that the proliferation of minor veins is a continuous trait, which can sustain a C_4_ cycle only over a threshold in this species. Since CO_2_ refixation by Rubisco takes place in the IS cells in C_4_ *A. semialata* (Ellis, 1974b; Ueno & Sentoku, 2006), the leaf anatomy of the hybrids might prevent an optimal coupling between carbon assimilation and reduction reactions, therefore decreasing the efficiency of the C_4_ pathway. The distribution of organelles among cell types was not quantified here, but a gene previously linked to the relocation of organelles to BS cells (Wang et al., 2017) is more highly expressed in C_4_ accessions and upregulated in some hybrids compared to their non‐C_4_ parents, which might suggest that organelles are partially relocated to their BS. The size of the OS cells might also play a role in creating an effective C_4_ cycle, as suggested by the large differences between C_3_ and C_4_ accessions (Figure 2e). Few other C_4_ species have an extra layer of BS cells outside of the BS cells containing chloroplasts, and the presence of an OS may represent an increased resistance to the C_4_ acid shuttle (Lundgren et al., 2014). In the grass genus *Neurachne* that presents a similar C_4_ anatomy, the smallest OS is also observed in C_4_ individuals, with intermediate species presenting intermediate OS cell sizes (Khoshravesh et al., 2020), supporting the idea that OS cells must be reduced during C_4_ evolution. Here, the OS cells were significantly reduced in the hybrids relative to their non‐C_4_ parents, with most C_3_+C_4_ × C_4_ individuals and a few C_3_ × C_4_ having OS cell sizes within the C_4_ range (Figure 2f), which suggests that this character might have played a minor role, if any, in reducing the efficiency of the C_4_ acid shuttle in the hybrids.

C_4_ activity could also have been limited by gene expression and protein activity. Despite significant increases in the transcript abundance of core C_4_ genes, particularly the genes encoding PEPC (Figure 4), it is possible that the insufficient expression levels of some genes might have reduced the efficiency of the C_4_ cycle, for example, by decreasing the rate of carboxylation and/or decarboxylation reactions. Furthermore, the lack of proper compartmentation of the expression/activity of these genes due to a non‐tissue‐specific expression of C_3_ or C_3_+C_4_ alleles could have impaired the formation of intermediate metabolite pools, therefore preventing the operation of an efficient C_4_ cycle (Brown & Bouton, 1993; Ermakova et al., 2021). Finally, the lack or insufficiency of posttranscriptional and posttranslational modifications might also have affected the realized enzyme activity. This might be particularly relevant in the case of hybrids, where the cell environment may be substantially modified by pleiotropic effects emerging from the expression of two divergent genomes. Detailed analyses of cellular localization and activity of C_4_ enzymes coupled to CO_2_‐labelling studies are necessary to identify the major determinants of the phenotype observed in the hybrids reported here.

### 4.2 Photosynthetic performance of plants expressing partial C_4_ traits and implications for the model of C_4_ evolution

The current model of C_4_ evolution assumes that the sequential acquisition of certain features, such as Rubisco activity in bundle sheath cells and upregulation of core C_4_ enzymes, increases photosynthetic output and consequently fitness in an environment that favours the C_4_ physiology (Heckmann et al., 2013; Malmmann et al., 2014; Monson & Moore, 1989; Sage, 2004). However, this Mt. Fuji‐like fitness landscape (Heckmann et al., 2013) is conditional on the presence of anatomical enablers; when these are lacking, the C_4_ phenotype is not accessible because intermediate states have lower fitness than the C_3_ state (Heckmann, 2016). The two groups of intraspecific hybrids generated in this study provide an opportunity to examine this prediction while controlling for phylogenetic effects, with C_3_ × C_4_ and C_3_+C_4_ × C_4_ plants being proxies for the upregulation of C_4_ traits at an early and intermediate stage, respectively, along C_4_ evolution. Here, we show that the CE of C_3_ × C_4_ hybrids is lower than that of both parents (Figures 4 and 5a), despite significant changes towards the C_4_ phenotype in leaf anatomy and gene expression. In C_3_+C_4_ × C_4_ hybrids however, CE was increased in comparison to the C_3_+C_4_ parent, and was not significantly different from C_4_ accessions. Such disparate effects of upregulating C_4_ components in a non‐C_4_ background are compatible with the initial prediction that fitness gains are conditional on the presence of enabling factors, which here might have been present in C_3_+C_4_ parents, but lacking in the C_3_ plants. Such changes would have acted as a switch that permitted a full C_4_ pathway to operate, and therefore increase photosynthetic performance. Note however that such gains in photosynthetic performance are restricted to conditions of low CO_2_ concentration in the leaf (Figure 4). In fact, at ambient CO_2_ levels, several individuals of both hybrid groups had lower photosynthetic rates than both parents, suggesting that the partial upregulation of C_4_ components, all at once, might have negative consequences, possibly due to pleiotropic effects (e.g., C_4_ enzymes competing for reducing power and ATP with other cell reactions). This in turn supports the idea that the changes towards a full C_4_ physiology must be built sequentially upon pre‐existing characteristics that enable the next change to provide a fitness advantage (Heckmann, 2016). The fertility of the F1 hybrids reported here is not known yet, although evidence of rare gene flow between photosynthetic types in the wild suggest that backcrossing is possible (Bianconi et al., 2020; Olofsson et al., 2016, 2021). If a F2 population can be produced, characterization of the photosynthetic performance of such individuals expressing C_4_ components independently, particularly under conditions expected to favour C_4_ plants, including long‐term exposure to low CO_2_ levels, should provide further insights on whether C_4_ evolution must follow a particular sequence of events for it to be viable.

## 5 CONCLUSIONS

In this study, we characterize the phenotype of hybrids between different photosynthetic types in the grass *A. semialata* to investigate how the upregulation of components of the C_4_ trait affects photosynthetic performance. We show that the hybrids have in most cases anatomical traits and gene expression patterns that are intermediate between those of the parents, and this leads to C_4_ activity that is equally intermediate between the two parents. The physiological benefits of a partial C_4_ metabolism in the hybrids appear only at low CO_2_ levels; at ambient CO_2_, there is no evidence of enhanced photosynthetic performance in the hybrids relative to their non‐C_4_ parents. Some hybrid individuals in fact perform worse than both parents at ambient CO_2_, and this possible hybrid depression could explain the lack of a clear hybrid zone in regions where the distributions of C_4_ and non‐C_4_ *A. semialata* lineages overlap. Overall, our results support the hypothesis that photosynthetic gains arising from the upregulation of C_4_ features are conditional on coordinated changes in leaf anatomy and biochemistry. Future studies with the F2 offspring, where the different C_4_ components can be segregated, will be able to pinpoint the key genetic and phenotypic changes that lead to fitness gains, providing a unique opportunity to experimentally test long‐standing hypotheses about the evolution of C_4_ photosynthesis.

## CONFLICTS OF INTEREST

The authors declare no conflicts of interest.

## Supporting information

Supporting information.

Supporting information.

Supporting information.

Supporting information.

Supporting information.

Supporting information.

## Data Availability

Newly generated RNA sequencing datasets were deposited in the NCBI SRA database under Bioproject PRJNA752516. Scripts used for the genotyping analysis are available at https://github.com/matheusbianconi/RNAgenotyping_hybrids
