# Supplementary material for: Upregulation of C4 characteristics does not consistently improve photosynthetic performance in intraspecific hybrids of a grass
Source: Plant Cell Environ. 2022 Mar 10;45(5):1398–411. doi: 10.1111/pce.14301 (PMC9314825; doi:10.1111/pce.14301)
Supplement: Supplementary file 1 — Supporting information. [file PCE-45-1398-s005.pdf]

Supplementary information for:

Upregulation of C_4 characteristics does not consistently improve photosynthetic performance in intraspecific hybrids of a grass

Matheus E. Bianconi, Graciela Sotelo, Emma V. Curran, Vanja Milenkovic, Emanuela Samaritani, Luke T. Dunning, Lígia T. Bertolino, Colin P. Osborne, Pascal-Antoine Christin

This supplementary information contains 9 figures and 12 tables. Tables are provided in five separate files: Tables S1-S6, S10-S12 ("Tables_SI.xlsx") and separate XLSX files for Tables S7, S8, S9 and S10.

Fig. S1. Leaf anatomical variables measured in this study.

Fig. S2. Principal component analysis on 11,988 genes from the chromosome-level genome assembly of *Alloteropsis semialata.*

Fig. S3. Transcript abundance in reads per million (RPM) of selected genes encoding core C_4 enzymes in *Alloteropsis semialata*: (A) aspartate aminotransferase (ASP-AT, gene *aspat-3P4*), (B) NADP-dependent malic enzyme (NADP-ME, gene *nadpme-1P4*), (C) phosphoenolpyruvate carboxykinase (PCK, gene *pck1P1_LGT*), (D) pyruvate phosphate dikinase (PPDK, gene *ppdk-1P2*), and (E-I) phosphoenolpyruvate carboxylase (PEPC) genes. Transcript abundance computed for *A. semialata* samples extracted from Dunning et al. (2019a) are shown on the right for each gene.

Fig. S4. Transcript abundance of selected gene families with a role in photorespiration (A-K), and the gene encoding the transcription factor GOLDEN2-like (L) in F1 hybrids and the parental photosynthetic types in *Alloteropsis semialata*. (A) 2-phosphoglycolate (2-PG) phosphatase (PGLP), (B-C) flavin mononucleotide (FMN)-dependent glycolate oxidase (GOX), (D) glutamate:glyoxylate aminotransferase (GGT), (E-H) glycine decarboxylase (GDC) complex proteins -H, -P, -T and -L, (I-J) serine hydroxymethyltransferase (SHMT), (K) glycerate 3-kinase (GLYK). Transcript abundance in reads per million mapped reads (RPM).

Fig. S5. Heat map of differentially expressed genes related to C_4 photosynthesis and photorespiration. Asterisks indicate significant DE genes ($p < 0.05$) for comparisons between hybrid types (C_3 x C_4 vs C_3+C_4 x C_4) and between each hybrid type and the C_4 type. Only DE genes with at least two-fold change and base count > 500 are shown. Count data was transformed using the VST function of DESeq2 and scaled by row. See table S9 for full gene annotation

Fig. S6. Photosynthetic response to intercellular CO_2 (*A*/*Ci*) of F1 hybrids and the parental photosynthetic types in *Alloteropsis semialata*. Panels contain all *A*/*Ci* curves collected for each accession, with individual curves coloured with different shades of grey. Accession name and

cross/photosynthetic type are indicated on the top left. Vertical dashed lines indicate the CO_2 compensation point of each curve.

Fig. S7. Leaf stomata variables. (A) Steady-state stomatal conductance (g_s) at 400 μmol mol^{-1} (n = 2-4 leaves per accession). (B) Stomatal density on the abaxial side of the leaves (n = 2 leaves per accession, with stomata counts averaged from 5 fields per leaf; field area = 0.38 mm^2). Data points on the right are the means per accession within each cross/photosynthetic type, and different lower-case letters indicate statistical differences between groups (ANOVA, $p < 0.05$ post-hoc Tukey HSD; $n \geq 3$).

Fig. S8. Leaf temperature variation during *A*/*Ci* curves. Data points are individual *A*/*Ci* measurements for each plant (left panels) or with individuals grouped into cross/photosynthetic types (right panels). (A) All measurements, and measurements collected at reference CO_2 (B) < 100 μmol mol^{-1}, (C) between 100 and 400 μmol mol^{-1}, and (D) > 400 μmol mol^{-1}. Arrows indicate outlier individuals that were removed for the analysis in Fig. S9.

Fig. S9. Photosynthetic performance of F1 hybrids and the parental photosynthetic types in *Alloteropsis semialata* after removing outlier accessions (i.e. with T_{leaf} 1°C above the median). (A) Maximum carboxylation efficiency (CE), (B) CO_2 compensation point (CCP), and steady-state (C) net photosynthetic rate (A_{400}), and (D) intrinsic water use efficiency (iWUE, A/g_s) at reference CO_2 = 400 μmol mol^{-1}.

Table S1. Sample information.

Table S2. Genotyping using PCR/Sanger-sequencing.

Table S3. List of primer sequences used for genotyping.

Table S4. RNA-seq markers selected for genotyping analysis I (photosynthetic type).

Table S5. RNA-seq markers selected for genotyping analysis II (pollen parent).

Table S6. Raw leaf anatomy data.

Table S7. Normalized transcript abundance for the co-ortholog gene set.

Table S8. Normalized transcript abundance for the *A. semialata* complete genome gene set.

Table S9. Differential expression analysis between F1 hybrids and the C_4 parental type.

Table S10. Gene ontology enrichment analyses on genes differentially expressed between F1 hybrids and the C_4 parental type.

Table S11. Raw A/Ci data.

Table S12. Estimated photosynthetic parameters and steady-state measurements.

Supplementary Methods

Global transcriptome analyses

We performed differential expression (DE) analyses using the raw counts obtained after mapping the RNA-seq datasets to the coding sequences extracted from the chromosome-level genome assembly of *A. semialata* (Dunning et al. 2019; see main text; total number of genes = 45,145). To detect genes that were differentially expressed between each of the hybrid types and the parental types, we used the R package *DESeq2* (Love et al. 2014). Due to the lack of sufficient replicates for the C_3 and C_3+C_4 parental types, we restricted our analyses to the comparisons between hybrid types (C_3 x C_4 vs C_3+C_4 x C_4), and between each of these and the C_4 type. Only genes with more than 10 counts across all accessions were retained for the DE analysis (total = 29,200 genes). For each comparison, we used a false discovery rate (FDR) of 0.05 as cut-off value. We then investigated whether each of the sets of DE genes were associated with any particular metabolic function using a gene ontology (GO) enrichment analysis with the R package *clusterProfiler* (Yu et al. 2012). We first used Orthofinder v2.5.2 (Emms and Kelly 2019) with default parameters to identify the corresponding orthologs of *A. semialata* in the closely related grass species *Setaria italica* (v2.2), *Sorghum bicolor* (v3.1.1) and *Oryza sativa* (v7.0; genomes extracted from Phytozome v13; Goodstein et al. 2012). We extracted the GO annotations for the three genomes using the Biomart tool of Phytozome 13, and transferred the annotations to the *A. semialata* gene set using the orthology information obtained from Orthofinder. We then performed a GO enrichment analysis on the DE gene set resulting from each of the three comparisons using the *enricher* function of *clusterProfiler* with a *p*-value cut-off of 0.05. The identity of C_4- and photorespiration-related genes in the genome of *A. semialata* was extracted from the annotations used in Bianconi et al. (2018) and Dunning et al. (2019).

Supplementary References

Bianconi ME, Dunning, LT, Moreno-Villena JJ, Osborne CP, Christin PA. 2018. Gene duplication and dosage effects during the early emergence of C_4 photosynthesis in the grass genus *Alloteropsis*. Journal of Experimental Botany, 69, 1967–1980.

Dunning LT, Olofsson JK, Parisod C, Choudhury RR, Moreno-Villena JJ, Yang Y, Dionora J, Paul Quick W, Park M, Bennetzen JL, et al. 2019. Lateral transfers of large DNA fragments spread functional genes among grasses. Proceedings of the National Academy of Sciences, USA 116: 4416–4425.

Emms DM, Kelly S. 2019. OrthoFinder: Phylogenetic orthology inference for comparative genomics. Genome Biology 20: 238.

Goodstein DM, Shu S, Howson R, Neupane R, Hayes RD, Fazo J, Mitros T, Dirks W, Hellsten U, Putnam N, et al. 2012. Phytozome: A comparative platform for green plant genomics. Nucleic Acids Research 40: D1178–D1186.

Love MI, Huber W, Anders S. 2014. Moderated estimation of fold change and dispersion for RNA-seq data with DESeq2. Genome Biology, **15**, 550.

Yu G, Wang L, Han Y, He Q. 2012. clusterProfiler: an R package for comparing biological themes among gene clusters. OMICS: A Journal of Integrative Biology, 16, 284-287.

Fig. S1

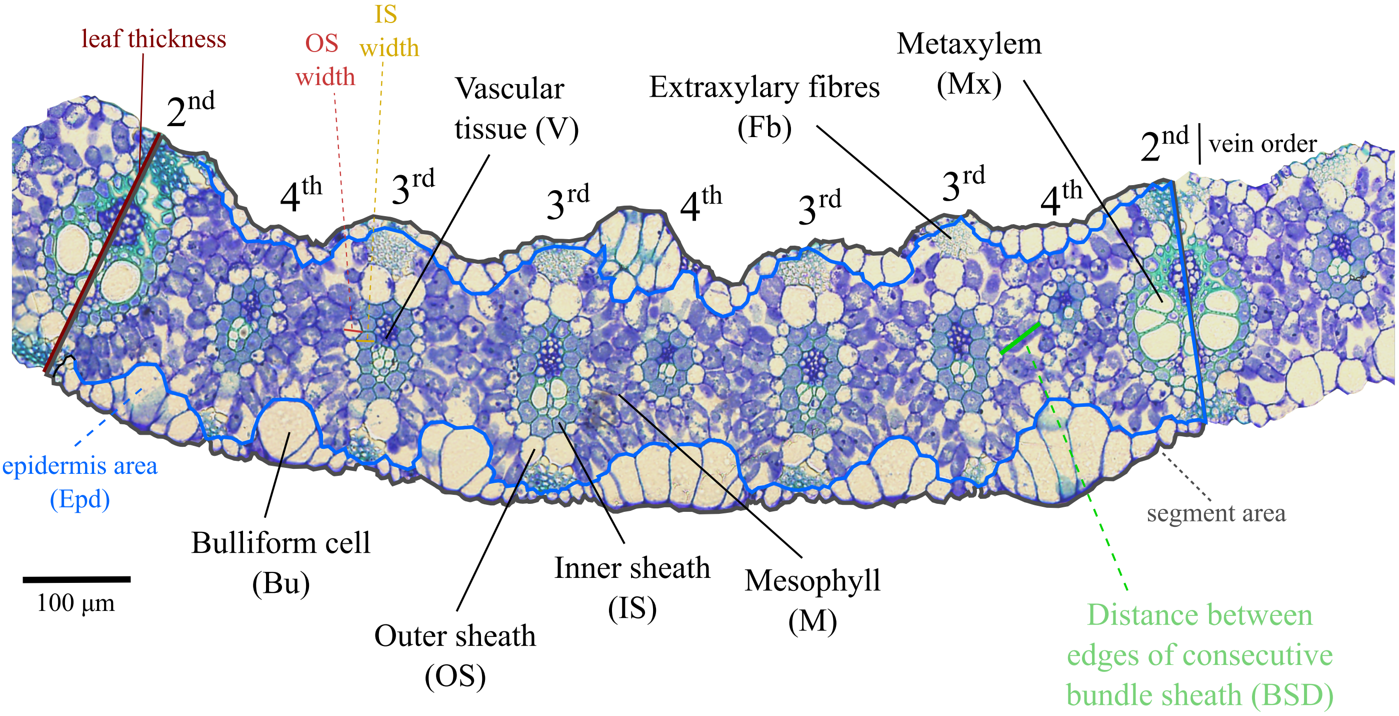

Fig. S1. Leaf anatomical variables measured in this study.

Fig. S2

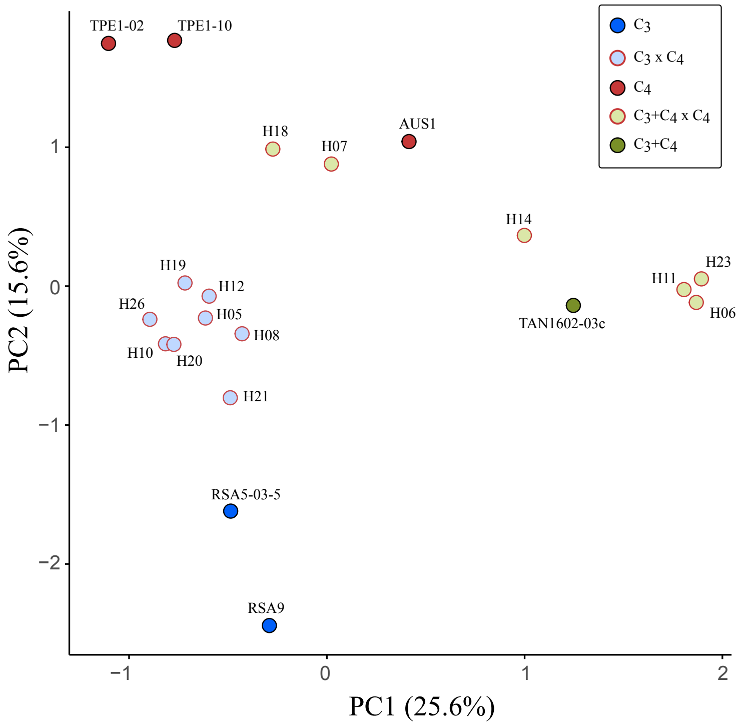

Fig. S2. Principal component analysis on 11,988 genes from the chromosome-level genome assembly of *Alloteropsis semialata*.

Fig. S3

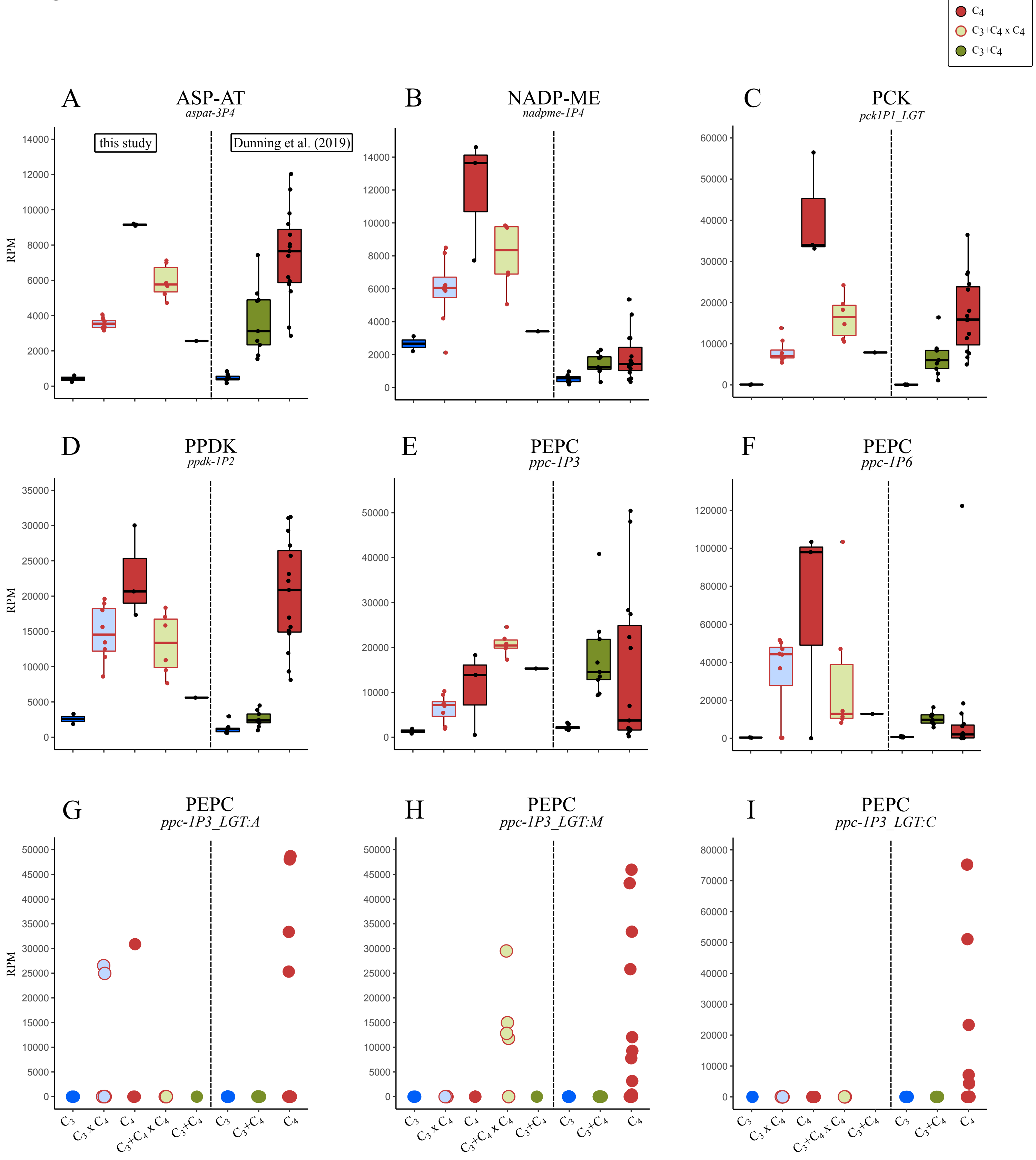

Fig. S3. Transcript abundance in reads per million (RPM) of selected genes encoding core C_4 enzymes in *Alloteropsis semialata*: (A) aspartate aminotransferase (ASP-AT, gene *aspat-3P4*), (B) NADP-dependent malic enzyme (NADP-ME, gene *nadpme-1P4*), (C) phosphoenolpyruvate carboxykinase (PCK, gene *pck1P1_LGT*), (D) pyruvate phosphate dikinase (PPDK, gene *ppdk-1P2*), and (E-I) phosphoenolpyruvate carboxylase (PEPC) genes. Transcript abundance computed for *A. semialata* samples extracted from Dunning et al. (2019a) are shown on the right for each gene.

Fig. S4

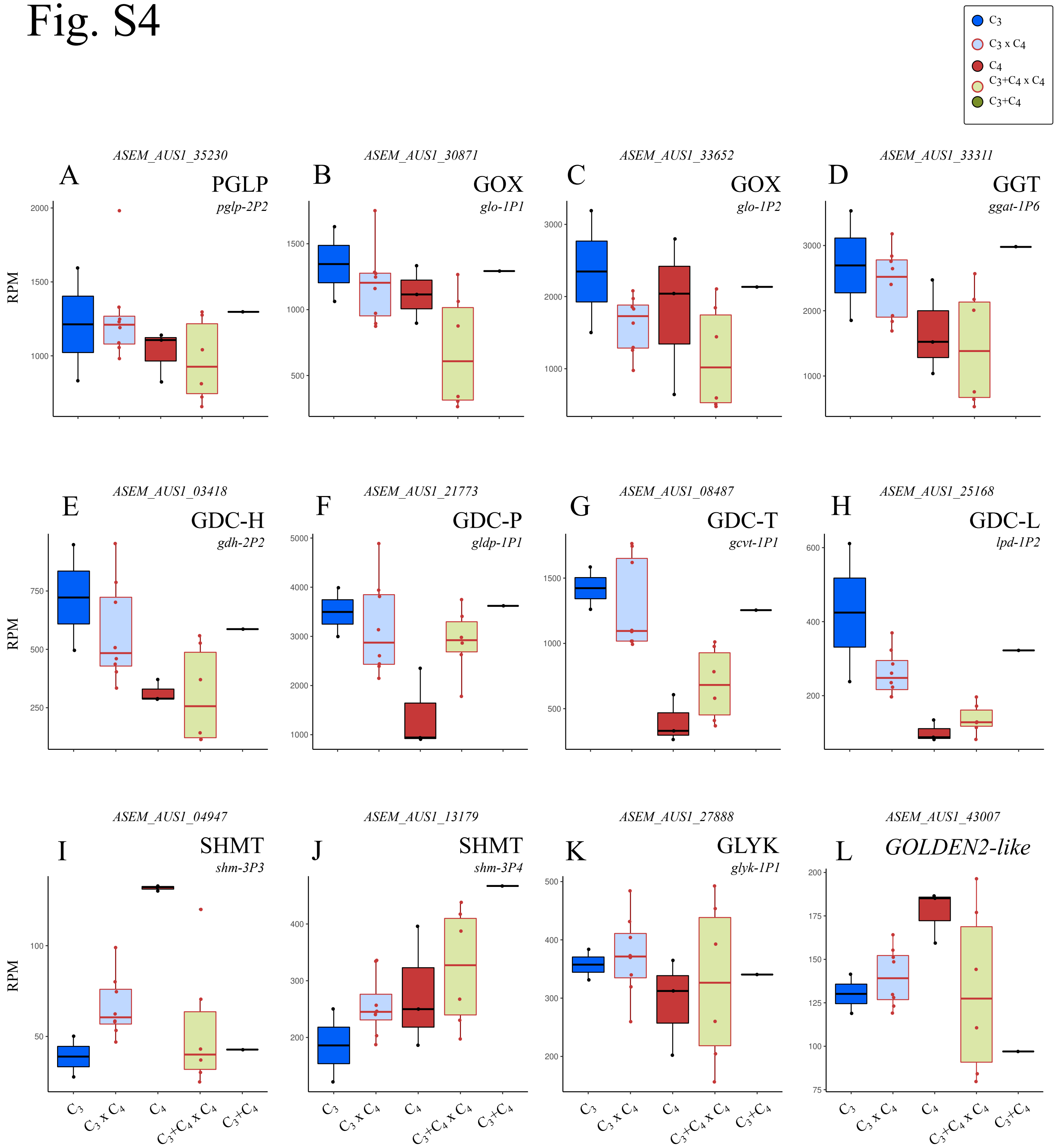

Fig. S4. Transcript abundance of selected gene families with a role in photorespiration (A-K), and the gene encoding the transcription factor *GOLDEN2*-like (L) in F1 hybrids and the parental photosynthetic types in *Alloteropsis semialata*. (A) 2-phosphoglycolate (2-PG) phosphatase (PGLP), (B-C) flavin mononucleotide (FMN)-dependent glycolate oxidase (GOX), (D) glutamate:glyoxylate aminotransferase (GGT), (E-H) glycine decarboxylase (GDC) complex proteins -H, -P, -T and -L, (I-J) serine hydroxymethyltransferase (SHMT), (K) glycerate 3-kinase (GLYK). Transcript abundance in reads per million mapped reads (RPM).

Fig. S5

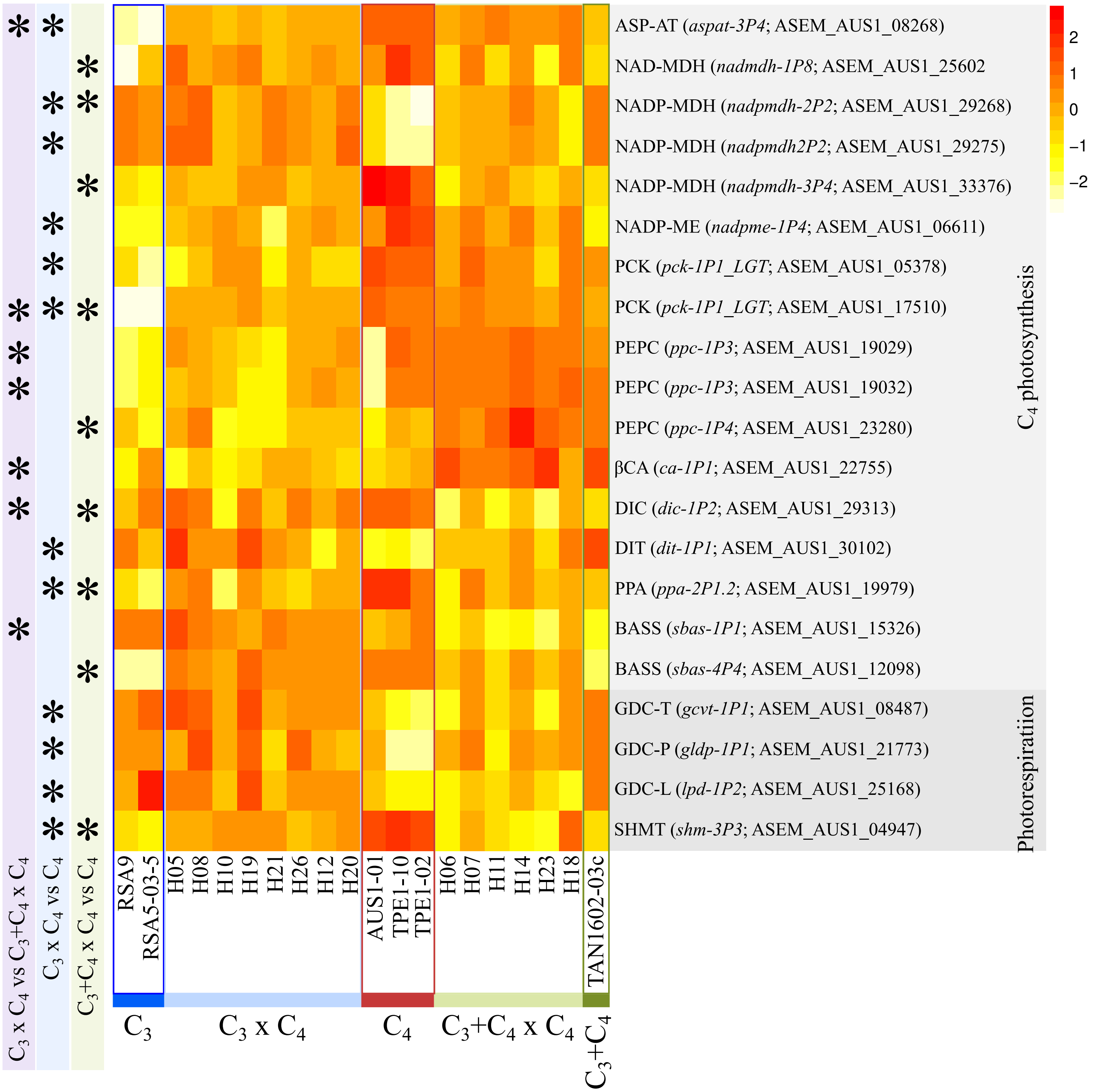

Fig. S5. Heat map of differentially expressed genes related to C_4 photosynthesis and photorespiration. Asterisks indicate significant DE genes ($p < 0.05$) for comparisons between hybrid types (C_3 x C_4 vs C_3+C_4 x C_4) and between each hybrid type and the C_4 type. Only DE genes with at least two-fold change and base count > 500 are shown. Count data was transformed using the VST function of DESeq2 and scaled by row. See table S9 for full gene annotation.

Fig. S6

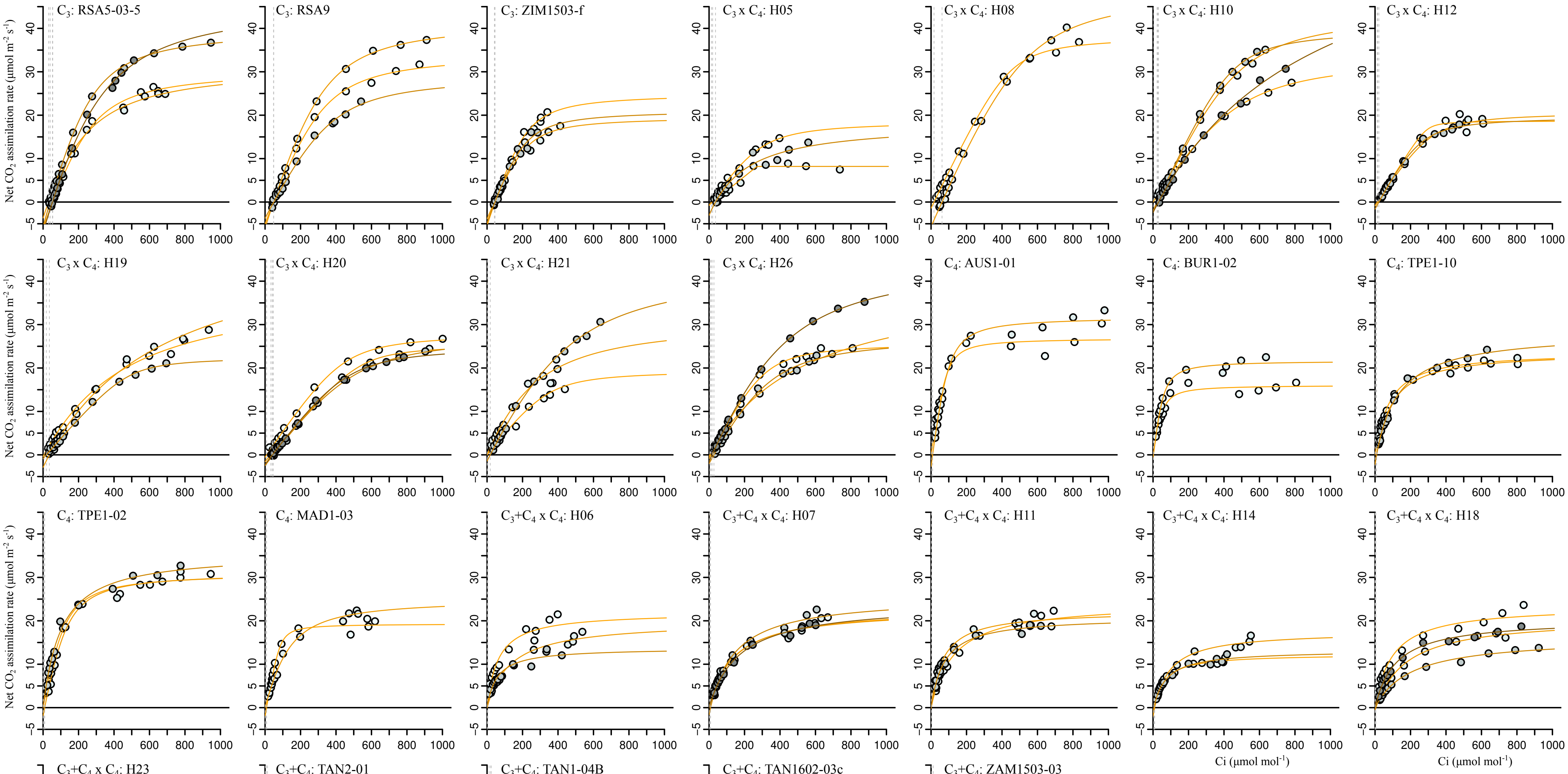

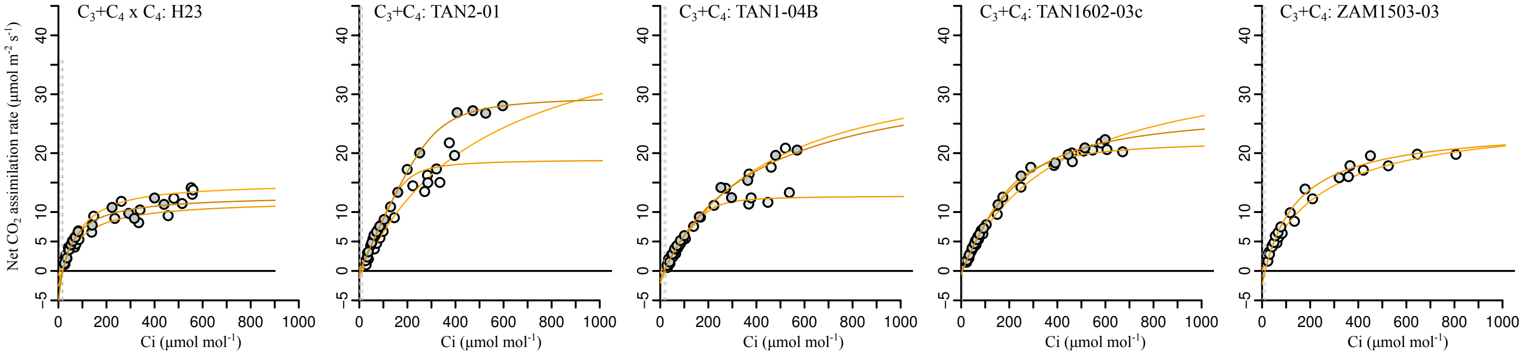

Fig. S6. Photosynthetic response to intercellular CO_2 (*A*/*Ci*) of F1 hybrids and the parental photosynthetic types in *Alloteropsis semialata*. Panelscontain all *A*/*Ci* curves collected for each accession, with individual curves coloured with different shades of grey. Accession name and cross/photosynthetic type are indicated on the top left. Vertical dashed lines indicate the CO_2 compensation point of each curve.

Fig. S7

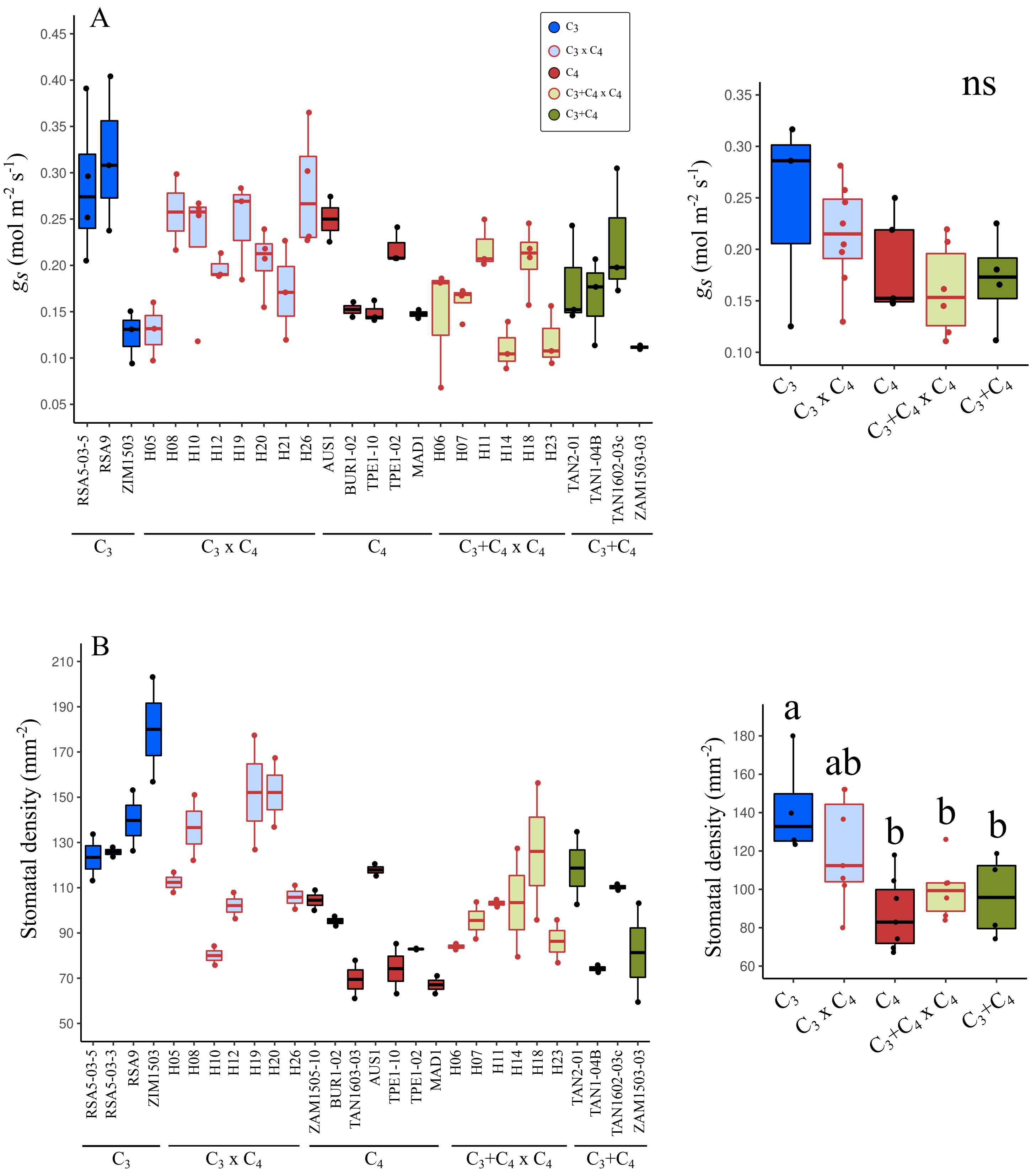

Fig. S7. Leaf stomata variables. (A) Steady-state stomatal conductance (g_s) at 400 µmol mol^{-1} (n = 2-4 leaves per accession). (B) Stomatal density on the abaxial side of the leaves (n = 2 leaves per accession, with stomata counts averaged from 5 fields per leaf; field area = 0.38 mm^2). Data points on the right are the means per accession within each cross/photosynthetic type, and different lower-case letters indicate statistical differences between groups (ANOVA, $p < 0.05$ post-hoc Tukey HSD; n ≥ 3).

Fig. S8

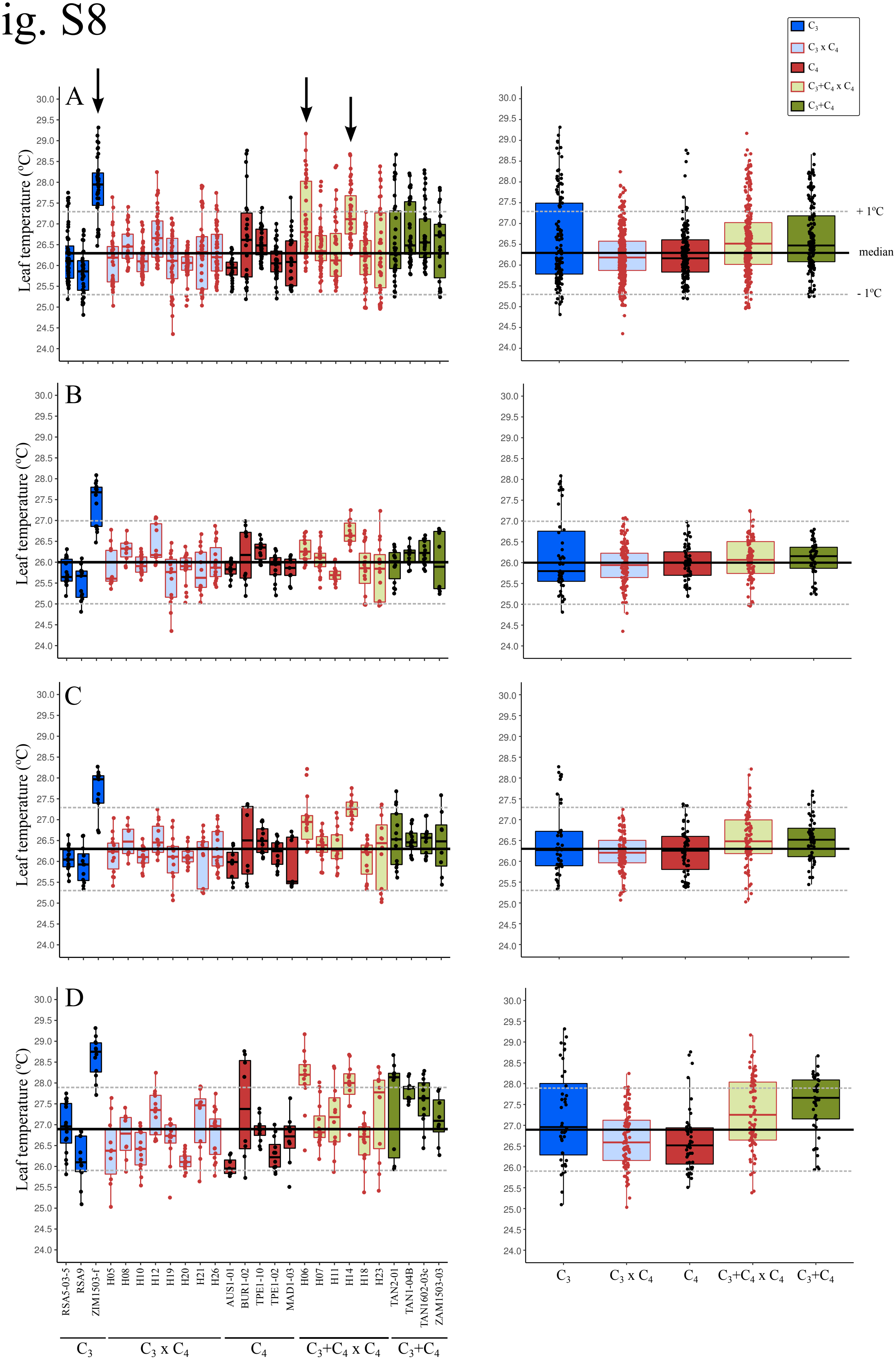

Fig. S8. Leaf temperature variation during A/Ci curves. Data points are individual A/Ci measurements for each plant (left panels) or with individuals grouped into cross/photosynthetic types (right panels). (A) All measurements, and measurements collected at reference CO_2 (B) < 100 µmol mol-1, (C) between 100 and 400 µmol mol^{-1}, and (D) > 400 µmol mol-1. Arrows indicate outlier individuals that were removed for the analysis in Fig. S9.

Fig. S9

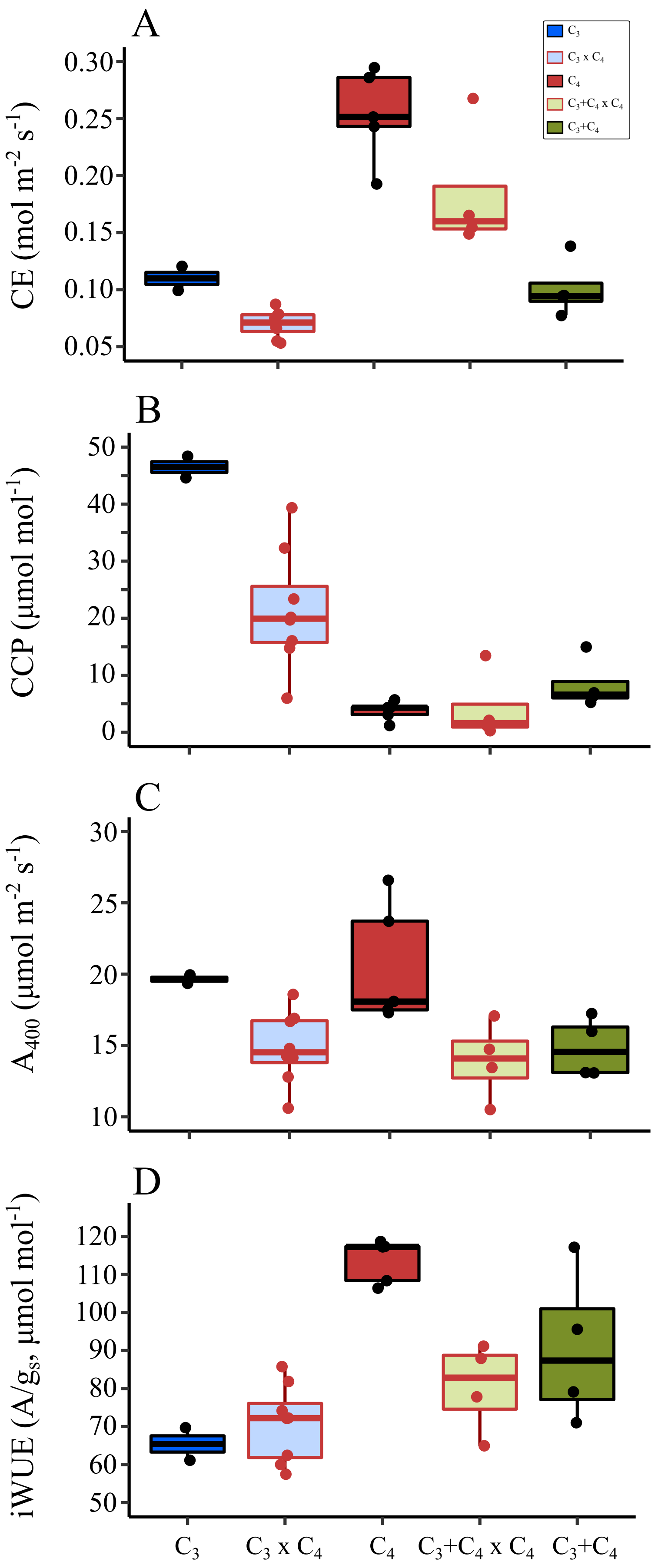

Fig. S9. Photosynthetic performance of F1 hybrids and the parental photosynthetic types in *Alloteropsis semialata* after removing outlier accessions (i.e. with T_{leaf} 1°C above the median). (A) Maximum carboxylation efficiency (CE), (B) CO_2 compensation point (CCP), and steady-state (C) net photosynthetic rate (A400), and (D) intrinsic water use efficiency (iWUE, *A*/*gs*) at reference CO_2 = 400 µmol mol^{-1}.
